# Functional, Sexual and Psychosexual Outcomes After Penile Fracture Repair: A Scoping Review

**DOI:** 10.7759/cureus.111941

**Published:** 2026-07-02

**Authors:** Althea O George, Sushmit Adhya, Lean Al Saqer, Mohamad Kokash, Olalekan Adeyeye, Akintayo J Akingbehin

**Affiliations:** 1 Urology, Royal Derby Hospital, Derby, GBR; 2 Urology, Royal London Hospital, London, GBR; 3 General Practice, Mercy and West Lancashire Teaching Hospitals NHS Trust, Southend, GBR; 4 Urology, Surgery Interest Group of Africa, Lagos, NGA; 5 Medicine and Surgery, Priory Hospital North London, London, GBR

**Keywords:** erectile dysfunction, false penile fracture, penile fracture, quality of life, rupture of corpora cavernosa, rupture of tunica albuginea, sexual function, surgical reconstruction

## Abstract

Penile fracture is a rare and underreported urological emergency, defined as traumatic rupture of the tunica albuginea of the corpus cavernosum. Most cases result from vigorous sexual intercourse or direct blunt trauma to an erect penis. While conservative management was once standard, high rates of long-term complications - including severe penile curvature, fibrotic plaques, and permanent erectile dysfunction (ED) - have led to a universal preference for immediate surgical repair. Although anatomical and physiological outcomes after surgery are well documented, the long-term psychosexual impact remains poorly characterised. Many patients report performance anxiety, fear of recurrence, and changes in sexual habits, but these experiences are often overlooked in favour of objective hemodynamic assessments.

This scoping review aims to map and synthesise current evidence on functional, sexual, and psychosexual outcomes after surgical repair of penile fractures. Specifically, it examines the impact of surgical timing on erectile function, identifies independent risk factors for postoperative ED, and evaluates psychological effects on sexual satisfaction, relationship dynamics, and ejaculatory function.

This review followed the Preferred Reporting Items for Systematic Reviews and Meta-Analyses Extension for Scoping Reviews (PRISMA-ScR) framework. Comprehensive searches were conducted in PubMed/MEDLINE, EMBASE, the Cochrane Library, SCOPUS, and Web of Science. Data charting included patient demographics, injury mechanisms, diagnostic methods, surgical timing, and validated psychometric assessments across nine core studies.

Immediate surgical intervention, ideally within 24 hours, is the gold standard and is associated with lower postoperative ED rates (6.5% to 16.5%) than delayed surgery (up to 42.9%) and conservative management (up to 52.9%). Independent predictors of poor outcomes include age over 50, urethral injury, bilateral corporal involvement, and tunical defects larger than 2 centimetres. Early repair reduces scarring and penile curvature. However, psychosexual outcomes remain complex. While erectile function is usually preserved, many patients experience psychogenic issues. Up to 77.5% report persistent fear of recurrence, and nearly 69% change their sexual habits. Changes in ejaculatory latency are also observed and correlate with higher depressive scores. Broad psychiatric screening tools do not show increased rates of clinical depression or generalised anxiety, indicating that psychological distress is specific to sexual performance and relationships.

Immediate surgical repair remains the standard approach for preserving erectile function and anatomical integrity after penile fracture. However, postoperative recovery should not be assessed solely by structural or hemodynamic outcomes. The available evidence suggests that some patients experience persistent fear of recurrence, avoidance behaviours, performance anxiety, and reduced sexual confidence after repair. These findings highlight the need for more consistent assessment of psychosexual outcomes in follow-up studies. Multidisciplinary postoperative support, including psychological counselling, sexual health assessment, and partner-centred care, may be considered where clinically appropriate, but further research is needed to define optimal follow-up and rehabilitation strategies.

## Introduction and background

Penile fracture is a severe urological emergency that demands immediate recognition, diagnosis, and surgical intervention to prevent permanent functional and psychosexual impairment [1]. It is defined as a traumatic, complete rupture of the tunica albuginea, the fibrous envelope surrounding the corpora cavernosa. The injury’s biomechanics depend on the penis’s physiological state at the time of trauma. In the flaccid state, the tunica albuginea is about 2 millimetres thick, providing significant resistance to bending, torsion, or blunt force [2].

During sexual arousal, the tunica albuginea stretches and thins to 0.25 to 0.5 millimetres as intracavernosal pressure rises. In this state, the penis becomes highly susceptible to abrupt trauma. Sudden blunt force can cause intracavernosal pressure to exceed 1500 mm Hg, overwhelming the tunica’s tensile strength and resulting in a tear with immediate blood extravasation into surrounding tissues [1, 3]. Penile fracture is a rare and likely underreported urological emergency, with reported incidence varying across populations, healthcare systems, and patterns of presentation. True rates are likely underreported due to embarrassment, stigma, and misdiagnosis. Most cases occur in men in their 30s and 40s, during peak sexual activity [4].

The causes of penile fracture vary by region and culture. In Western countries, most cases result from sexual intercourse, especially during vigorous thrusting or high-risk positions where control is limited [5]. In some Middle Eastern and North African regions, self-inflicted injury through forceful bending of the erect penis (*taqaandan*) is common, often to conceal an erection [6]. This practice leads to many non-coital fractures in these areas.

Penile fracture typically presents with an audible "popping" or snapping sound, sudden pain, and immediate loss of erection. Blood extravasates into subcutaneous tissues, causing rapid swelling, bruising, and deviation of the penis, known as the "eggplant deformity." In 10% to 38% of severe cases, the injury extends to the corpus spongiosum and urethra, leading to symptoms such as blood at the meatus, haematuria, dysuria, or urinary retention [7].

Historically, penile fractures were managed conservatively with compressive dressings, ice, antibiotics, anti-inflammatories, and medications to suppress erections. However, these approaches led to high rates of severe complications, including uncorrectable curvature, painful plaques, and permanent erectile dysfunction. As a result, conservative management has been replaced in favour of surgical intervention [4, 8]. Immediate surgical exploration is now the standard of care. Surgery involves evacuating the haematoma, identifying and repairing the tunical defect, removing damaged tissue, and closing the defect with fine, absorbable sutures. The goal is to preserve cavernosal hemodynamics and anatomical integrity to maintain erectile function [1, 9].

Although timely surgical repair is widely accepted, the definition of clinical success remains limited. Current assessments primarily focus on structural and functional outcomes, including penile curvature, pain, and erectile function, often measured using validated tools such as the International Index of Erectile Function-5 (IIEF-5) [10] or Sexual Health Inventory for Men (SHIM) score [11]. However, sexuality also involves psychological well-being, and traumatic genital injuries may cause distress such as performance anxiety, fear of recurrence, and psychogenic sexual dysfunction [12]. Despite these psychological risks, psychosexual outcomes after penile fracture repair remain underexplored in the literature. However, anatomical and physiological outcomes, including penile morphology, pain, erectile function, and haemodynamic recovery, are also important markers of postoperative success.

Therefore, this scoping review aims to provide a comprehensive overview of postoperative outcomes after penile fracture repair, integrating structural, functional, sexual, and psychosexual domains.

## Review

Methods

The methodology adhered to the Preferred Reporting Items for Systematic Reviews and Meta-Analyses extension for Scoping Reviews (PRISMA-ScR) guidelines [13]. A review protocol was developed to guide the search strategy, eligibility criteria, study selection, and data extraction process; however, it was not prospectively registered in a public review registry. The literature search was last conducted on May 29, 2026.

Information Sources and Search Strategy

Systematic electronic searches were carried out across PubMed/MEDLINE, Embase, the Cochrane Library, Scopus, and Web of Science. The included articles spanned publications from 2011 to 2026. Search strategies combined MeSH terms and free-text keywords, including ‘penile fracture’, ‘fracture of penis’, ‘rupture of corpora cavernosa’, ‘rupture of tunica albuginea’, ‘surgical repair’, ‘immediate repair’, ‘delayed repair’, ‘erectile dysfunction’, ‘sexual function’, ‘psychosexual’, ‘anxiety’, ‘depression’, ‘quality of life’, ‘IIEF-5’, and ‘SHIM’, using Boolean operators to maximise sensitivity and maintain contextual relevance.

The core search strategy was built upon three primary conceptual blocks and detailed in Table 1.

**Table 1:** Search strategy IIEF: International Index of Erectile Function; SHIM: Sexual Health Inventory for Men

Search component	Keywords/search terms	Boolean operators/notes
Concept 1: Condition	“penile fracture” OR “fracture of penis” OR “rupture of corpora cavernosa” OR “rupture of tunica albuginea” OR “false penile fracture”	OR
Concept 2: Intervention/management	management OR treatment OR surgery OR “surgical reconstruction” OR “surgical repair” OR “immediate repair” OR “delayed repair”	OR
Concept 3: Outcomes	outcomes OR “erectile dysfunction” OR “sexual function” OR psychosexual OR psychological OR anxiety OR depression OR “partner satisfaction” OR “quality of life” OR IIEF OR IIEF-5 OR SHIM OR “Sexual Health Inventory for Men”	OR
Combined Boolean search string	(“penile fracture” OR “fracture of penis” OR “rupture of corpora cavernosa” OR “rupture of tunica albuginea” OR “false penile fracture”) AND (management OR treatment OR surgery OR “surgical repair” OR “immediate repair” OR “delayed repair”) AND (outcomes OR “erectile dysfunction” OR “sexual function” OR psychosexual OR psychological OR anxiety OR depression OR “quality of life” OR IIEF OR IIEF-5 OR SHIM)	AND

Data Charting and Outcome Measures

Data extraction included patient demographics, injury mechanisms, diagnostic methods, surgical timing, operative approach, anatomical outcomes, and postoperative sexual function outcomes. Where available, erectile and sexual function outcomes were extracted using validated tools such as the International Index of Erectile Function-5 (IIEF-5) and the Sexual Health Inventory for Men (SHIM), alongside reported erectile dysfunction rates, penile curvature, painful erections, ejaculatory outcomes, and psychosexual assessment measures.

Outcome Domains and Assessment Tools

Postoperative outcomes were grouped into anatomical, sexual, psychological, and relationship domains. Sexual function outcomes included erectile function, erectile dysfunction severity, intercourse satisfaction, overall sexual satisfaction, ejaculation-related outcomes, and ability to resume sexual activity. Where reported, erectile function was assessed using validated tools such as the International Index of Erectile Function-5 (IIEF-5) or Sexual Health Inventory for Men (SHIM) [10, 11]. Broader sexual function was assessed using reported measures such as Erection Hardness Score, ejaculatory latency, painful erections, sexual satisfaction, and changes in sexual activity.

Psychological outcomes included fear of recurrence, performance anxiety, depressive symptoms, general anxiety, and psychogenic erectile dysfunction. These were extracted and assessed using validated tools such as the Hospital Anxiety and Depression Scale (HADS) [14], Beck Depression Inventory [15], or study-specific psychosexual questionnaires.

Relationship outcomes included partner-related concerns, relationship satisfaction, self-esteem, sexual confidence, avoidance of intercourse, and changes in sexual habits or preferred sexual positions. Where available, these were extracted using instruments such as the Golombok-Rust Inventory of Sexual Satisfaction (GRISS) [16], Premature Ejaculation Diagnostic Tool (PEDT) [17], and Self-Esteem and Relationship (SEAR) questionnaire [18].

A detailed summary of the postoperative outcome domains, their components, and the validated assessment tools used across the included studies is provided in the Appendices.

Eligibility Criteria and Data Selection

Studies were selected using strict eligibility criteria to ensure inclusion of evidence specifically addressing long-term outcomes following surgical management of penile fracture. Eligible studies included prospective or retrospective cohort studies, multicentre studies, systematic reviews, and meta-analyses involving adult males with clinically or radiologically confirmed traumatic penile fracture. Included studies had to assess surgical exploration and repair, including comparisons of immediate versus delayed surgery or historical conservative treatment, and report functional or sexual outcomes after a minimum follow-up of six months. Key outcomes included erectile dysfunction, IIEF-5/SHIM scores, penile curvature, tunical scarring, painful erections, ejaculatory outcomes, and validated psychological or sexual function measures such as HADS, Beck Depression Inventory, GRISS, PEDT, and SEAR.

Studies were excluded if they involved fewer than five patients, animal or cadaveric models, narrative reviews, editorials, or non-peer-reviewed commentaries. Reports involving penetrating penile trauma, amputation, or degloving injuries were also excluded because these differ pathophysiologically from penile fracture. Studies evaluating only conservative management without a surgical comparator, those lacking long-term functional or sexual outcomes, follow-up shorter than six months, or non-English articles without certified translations were excluded.

Study Selection

**Figure 1:**
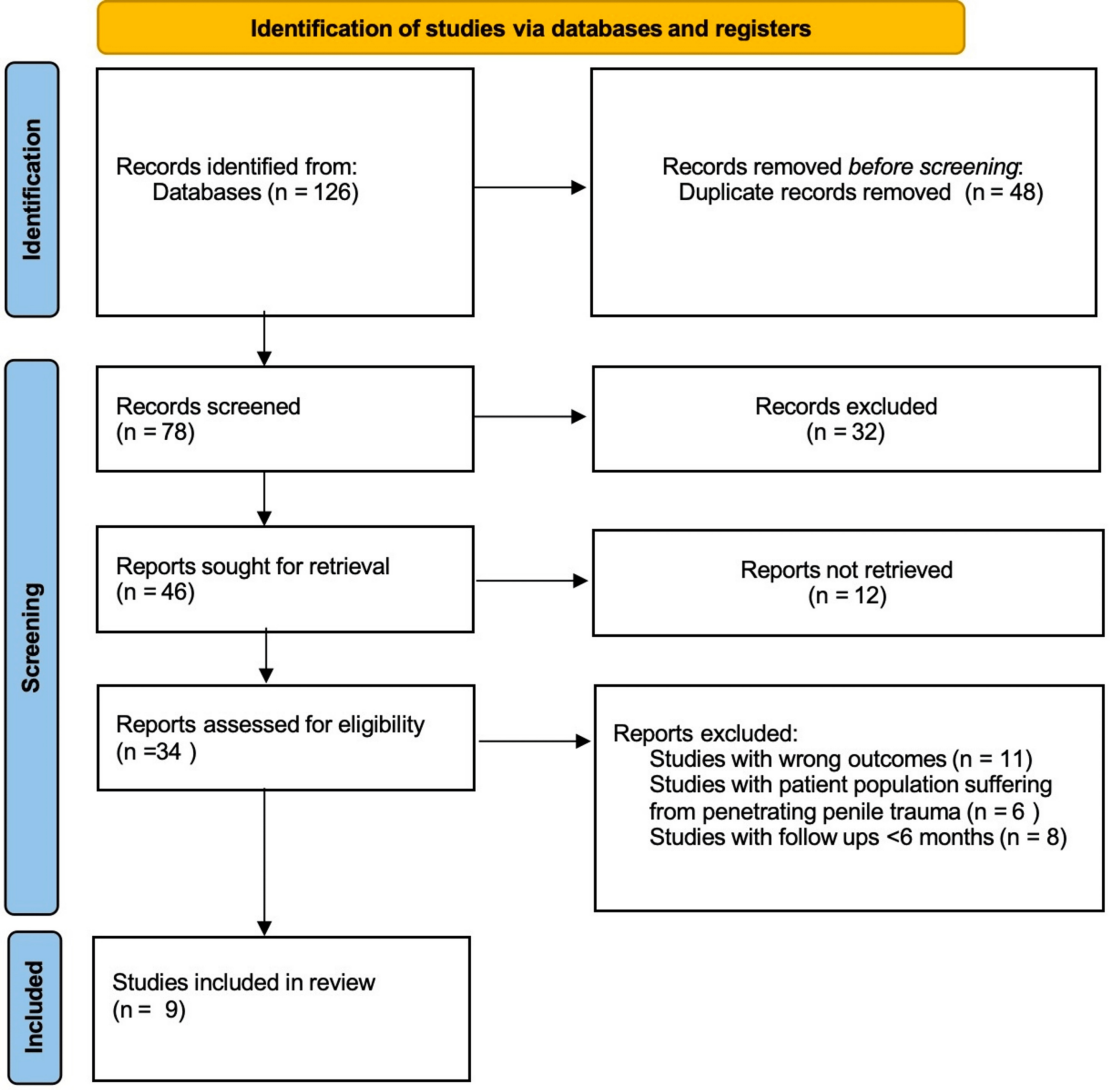
PRISMA flow diagram PRISMA: Preferred Reporting Items for Systematic Reviews and Meta-Analyses

A total of 126 records were identified through database searches. After the exclusion of 48 duplicate records, 78 records proceeded to title and abstract screening. Of these, 32 records were excluded because they were irrelevant to penile fracture repair outcomes, wrong population, wrong injury type, or review/editorial/case report, leaving 46 reports to be retrieved. Twelve reports could not be retrieved because full-text articles were unavailable or inaccessible at the time of screening, and 34 full-text reports were assessed for eligibility. Following full-text assessment, 25 reports were excluded: 11 due to inappropriate outcomes, six because the study population involved penetrating penile trauma, and eight because follow-up duration was less than six months. Ultimately, nine studies met the eligibility criteria and were included in the review. The flow of study selection is detailed in the PRISMA flow diagram below (Figure 1).

Study Characteristics and Patient Demographics

Application of the PRISMA-ScR eligibility criteria resulted in the selection of nine core studies for this scoping review, representing a dataset of over 3,900 patients worldwide. To give a detailed overview of the evidence landscape synthesised in this review, Table 2 presents the characteristics of the nine included studies.

**Table 2:** Study characteristics ED: erectile dysfunction; HADS: Hospital Anxiety and Depression Scale; GRISS: Golombok-Rust Inventory of Sexual Satisfaction; PEDT: Premature Ejaculation Diagnostic Tool

Primary author	Year	Study design	Sample size (N)	Primary focus/key outcomes evaluated
el-Assmy et al. [19]	2011	Retrospective cohort	180	Evaluated impact of delayed presentation (>24h), identifying advanced age and tunical tear size as primary ED risk factors.
Penbegul et al. [20]	2012	Controlled study	32 (vs. 30 controls)	Evaluated long-term psychological status (HADS, GRISS, PEDT); found no significant differences in generalised anxiety or depression versus controls.
De Luca et al. [21]	2017	Retrospective cohort	76	Tertiary referral centre experience evaluating long-term functional outcomes following immediate repair.
Bolat et al. [22]	2017	Retrospective cohort	64	Assessed psychosocial status and sexual function; noted progressive decline in relationship and self-esteem scores over 24 months.
Bozzini et al. [23]	2018	Retrospective multicentre	137	Evaluated impact of surgical delay (>8 hours) across 7 European centres, demonstrating significantly increased ED with delayed intervention.
Barros et al. [24]	2019	Prospective observational	58	Psychosexual impact: 77.5% feared recurrence, 68.9% altered sexual habits; performance anxiety linked to ED.
Kati et al. [25]	2019	Retrospective cohort	56	Investigated early surgical repair effects specifically regarding the preservation of erectile function.
Abdelrasheed et al. [4]	2024	Systematic review & meta-analysis	3,213	Immediate (<24h) vs. Delayed management; Comprehensive ED risk factors and incidence rates.
Shah et al. [26]	2026	Retrospective cohort	32	Investigated if mere surgical repair is sufficient; found 65% of patients manifested varying degrees of sexual dysfunction despite successful physical repair.

Data Synthesis

The data synthesis (Table 3) aggregates the core functional, anatomical, and psychosexual outcomes identified across the nine included studies.

**Table 3:** Data synthesis ED: erectile dysfunction

Outcome category	Synthesised clinical findings	Key contributing studies
Anatomical repair	Immediate intervention (<24h) significantly minimises residual penile curvature (1.8%) and structural deformities compared to delayed management.	Abdelrasheed et al., Bozzini et al. [4, 23]
Erectile function	Timely surgical repair reduces post-fracture organic erectile dysfunction rates to 6.5% - 16.5%, averting the >45% rates seen historically.	Kati et al., De Luca et al. [21, 25]
ED risk factors	Independent predictors of permanent ED include age >50 years, bilateral cavernosal tears, tunical defects >2cm, and urethral involvement.	Abdelrasheed et al., el-Assmy et al. [4, 19]
Psychosexual impact	Deep psychological trauma persists despite structural repair; 77.5% experience fear of recurrence, and roughly 65% to 69% experience habit alteration or varying degrees of sexual dysfunction.	Barros et al., Bolat et al., Shah et al. [22, 24, 26]
Psychiatric indices	Broad psychiatric morbidity (e.g., generalised anxiety or clinical depression) does not significantly increase; the distress is localised strictly to sexual contexts.	Penbegul et al. [20]

Results

Demographics and Aetiology Discovered Across the Cohorts

In the pooled analysis by Abdelrasheed et al., the mean patient age was 35.6 years (range: 19-72 years), with sexual intercourse as the leading cause of injury (48%), followed by manual manipulation or forced flexion (39%) [4]. In the South American prospective cohort by Barros et al., the mean age at presentation was 38.5 years, with most patients presenting with single-corporal tears [24].

Regional variations in aetiology were observed. The European multicentre cohort by Bozzini et al. reported sexual trauma (forceful thrusting and coital slips) as the predominant mechanism (80%) [23]. In contrast, Middle Eastern cohorts, such as that of el-Assmy et al., identified a higher proportion of injuries from non-coital mechanisms, particularly self-directed forced bending to suppress erections (*taqaandan*) [19]. Severe trauma characteristics, including concomitant urethral damage, were present in 16% of the Turkish cohort and up to 38% in select global populations [4, 25].

Diagnostic Pathways and Operative Techniques

The selected studies offered important findings on the influence of preoperative diagnostic pathways and intraoperative decisions on long-term structural and functional outcomes.

Discoveries on Non-invasive Diagnostics and Urethral Evaluation

In terms of diagnostics, the European Association of Urology (EAU) trauma guidelines cited across the studies suggest prioritising clinical judgment and recommending that typical presentations do not require delay in extensive imaging [27]. However, in equivocal scenarios, the meta-analysis by Abdelrasheed et al. noted that high-resolution magnetic resonance imaging (MRI) has a 100% positive predictive value for localising tunical defects, whereas ultrasonography is highly operator-dependent [4].

For urethral evaluation, if urethral injury is suspected based on blood at the meatus or haematuria, the guidelines strongly recommend intraoperative flexible cystoscopy over preoperative retrograde urethrography. This is because retrograde urethrography has been shown to yield high rates of false-positive results and to unnecessarily delay time-critical access to the operating room [27].

Discoveries on Incision Selection and Suture Materials

The surgical approach is mainly determined by the location of the tear. De Luca et al. reported using a circumferential subcoronal degloving incision as the standard approach in 76 consecutive patients, noting that it provides comprehensive exposure of both corpora cavernosa and the spongiosum, which is necessary for identifying concealed bilateral or urethral tears [21]. During tumescence, the tunica albuginea thickness decreases from approximately 2 millimetres in the flaccid state to 0.25-0.5 millimetres, making surgical exposure particularly delicate [1].

Conversely, el-Assmy et al. discovered that the choice of incision should be tailored based on the lesion's site along the shaft [19]. They determined that while a small, localised skin incision directly over the palpable defect is highly effective for distal unilateral tears to minimise post-circumcision oedema, proximal shaft ruptures should always be explored via a subcoronal degloving incision to minimise the risk of residual curvature [19].

With respect to suture selection, the literature strongly advises against the use of non-absorbable sutures. Abdelrasheed et al. and other surgical reviews concluded that not using fine, absorbable sutures (typically 2-0 or 3-0 polyglactin) with buried knots is independently associated with permanent, painful subcutaneous nodules and chronic localised inflammation [4].

Anatomical and Structural Outcomes

The primary immediate goals of surgical intervention are the thorough evacuation of the organising haematoma and the restoration of the tunica albuginea's structural integrity. The synthesised data overwhelmingly favours immediate surgical repair for the preservation of anatomical form [1, 28].

The specific timing of the surgery plays a statistically significant role in structural outcomes. In the comprehensive meta-analysis conducted by Abdelrasheed et al., the incidence of postoperative penile curvature significantly favoured immediate intervention (executed within 24 hours) [4]. Immediate repair cohorts demonstrated a clinical curvature rate of only 1.8%, compared to 4.5% in delayed groups (OR 0.33, p=0.034) [4].

This time-dependence is further supported by Barros et al., who found that 13.7% of patients developed postoperative penile curvature, which was significantly correlated with a delayed presentation exceeding 24 hours [24]. Additionally, Shah et al. reported a postoperative curvature rate of 12.5% at follow-up, noting that while mild deviations did not mechanically prevent vaginal penetration, they still caused considerable distress to the patients [26]. Kati et al. observed excellent structural outcomes with early repair, noting 0% curvature and a 3.5% rate of palpable nodules, though massive preoperative haematomas did predict transient postoperative pain [25].

Regarding surgical approach, De Luca et al. evaluated 76 consecutive patients and demonstrated that a circumferential subcoronal degloving incision provides optimal panoramic exposure of both corpora cavernosa and the spongiosum, which is crucial for identifying complex or hidden tears and preventing subsequent deformities [21].

Discoveries on Penile Curvature and Deformity

The meta-analysis by Abdelrasheed et al. compared the incidence of postoperative anatomical complications between early and delayed repair [4]. They discovered that immediate surgical intervention, typically performed within 24 hours of injury, yielded a significantly lower rate of postoperative penile curvature (1.8%) than delayed cohorts (4.5%; OR: 0.33, 95% CI: 0.12-0.92, p=0.034) [4].

This time dependence was further elucidated by findings from individual cohorts. In the prospective evaluation by Barros et al., 13.7% of patients developed mild postoperative penile curvature at 18 months, which was significantly correlated with a delayed presentation exceeding 24 hours [24].

Similarly, Shah et al. reported a postoperative curvature rate of 12.5% (four out of 32 patients) at their follow-up, discovering that while these mild deviations did not physically prevent vaginal penetration, they still caused considerable distress to the patients [26]. De Luca et al. discovered in their cohort that although mild residual angulation may occur postoperatively, it rarely requires subsequent surgical plication unless associated with extensive, unevacuated haematomas [21].

Discoveries on Tunical Scarring and Palpable Plaques

The formation of palpable tunical scars or plaques at the repair site is a common postoperative sequela. The systematic review by Abdelrasheed et al. revealed that the incidence of tunical scarring did not differ statistically between immediate and delayed surgical cohorts (5.4% vs. 4.5%; OR: 0.59, p=0.393), suggesting that scarring is primarily a function of the local healing response rather than surgical timing [4].

Shah et al. reported that 6.25% of their cohort (2 out of 32 patients) perceived a palpable, firm nodule at the site of suture placement, noting that burying the suture knots is a key intraoperative technique to prevent this occurrence [26]. In the cohort analysed by Kati et al., only one patient (1.7%) developed serious skin necrosis, demonstrating that early surgical debridement and evacuation of the highly pressurised haematoma effectively prevent tissue ischaemia and extensive scarring [25].

Sexual Outcomes and Organic Erectile Dysfunction

Erectile dysfunction remains the most feared and physically disabling organic complication following a penile fracture [29].

Abdelrasheed et al. established a clear threshold for organic recovery, discovering that immediate surgical repair within 24 hours restricts the overall postoperative ED rate to 6.6%-16.5%, whereas historical conservative management leads to catastrophic ED rates of 45.5%-52.9% [4]. The mean postoperative IIEF-5 scores in immediate surgery groups consistently remained high (20.5-22.5) [4].

The risk of delay was specifically quantified by Bozzini et al., who evaluated 137 patients across seven European centres [23]. They discovered that delaying surgical repair beyond a strict eight-hour window from the time of trauma resulted in a substantial and significant increase in postoperative ED rates (40.0% in delayed vs. 17.5% in early cohorts; OR: 3.1, p=0.003) [23]. Conversely, Kati et al. confirmed that when early surgical intervention is achieved, there is no statistically significant difference between preoperative and six-month postoperative IIEF-5 scores [25].

Impact of Treatment Modality and Surgical Timing

The primary timing analysis conducted by Abdelrasheed et al. established a clear, time-dependent threshold for organic recovery [4]. They discovered that immediate surgical repair within 24 hours of injury limits the overall postoperative ED rate to 6.6% to 16.5%, whereas conservative management leads to a catastrophic ED rate of 45.5% to 52.9% [4]. Furthermore, the mean post-exploration IIEF-5 scores in the immediate-surgery groups ranged from 20.5 to 22.5, whereas the conservative and delayed management cohorts scored significantly lower, ranging from 14.9 to 17.2 [4].

Bozzini et al. analysed 137 patients across seven European centres and discovered that delaying surgical repair beyond a strict eight-hour window from the time of trauma resulted in a substantial increase in postoperative ED rates (40.0% in delayed vs. 17.5% in early cohorts; OR: 3.1, 95% CI: 1.5-6.6, p=0.003) [23].

Kati et al. evaluated 56 patients who underwent early surgical repair (mean time to surgery of 10.5 hours) and found no statistically significant difference between preoperative and six-month postoperative IIEF-5 scores, confirming that early surgical intervention preserves the organic erectile mechanism [25].

Conversely, el-Assmy et al. followed up 180 patients and discovered that even with successful surgery, a delayed presentation beyond 24 hours remained a major hazard, with 13.3% of their delayed group experiencing severe difficulty maintaining an erection, which necessitated the regular use of sildenafil [19].

Independent Risk Factors for Postoperative Erectile Dysfunction

The selected studies identified several clinical and anatomical parameters that independently predict organic ED following surgical repair.

With respect to bilateral corporal involvement, Abdelrasheed et al. noted that bilateral corporal rupture carries a high risk of postoperative erectile dysfunction [4]. At the same time, De Luca et al. found that patients with bilateral injuries had an erectile dysfunction rate of 50.0% compared with only 10.3% in unilateral cases (OR: 8.7, 95% CI: 2.0-37.8, p=0.004) [4, 21]. 

Concomitant urethral injury also represents a critical determinant; el-Assmy et al. analysed 18 patients with concomitant urethral injuries and discovered that their postoperative erectile dysfunction rate was 28.6%, compared to only 4.2% in patients with intact urethras (OR: 9.1, 95% CI: 3.2-25.8, p<0.001) [19]. Furthermore, a large tunical tear size serves as an independent predictor of poor outcomes, as De Luca et al. discovered that a tunical defect exceeding 2 centimeters was independently associated with a significantly higher rate of postoperative erectile dysfunction, affecting 35.7% of patients compared to 9.5% of those with smaller defects (OR: 5.3, 95% CI: 1.5-18.7, p=0.010) [19, 21].

Advanced patient age is another major predictor; Abdelrasheed et al. identified age greater than 50 years as a major predictor of postoperative erectile dysfunction with an overall risk ratio of 1.65 (95% CI: 1.14-2.39), while el-Assmy et al. found that patients aged 40 years or older had a significantly higher risk of postoperative erectile dysfunction than younger patients, with rates of 11.1% versus 3.7% respectively (OR: 3.3, 95% CI: 1.1-9.8, p=0.028) [4, 19]. Pre-existing comorbidities and clinical presentation are similarly influential; Kati et al. discovered that the presence of a concomitant, unevacuated haematoma significantly elevated the risk of erectile dysfunction to 15.6% compared to 0% in patients without a haematoma (p=0.023), as the organising clot promotes localised cavernosal fibrosis [25]. Finally, concerning etiological variables, Shah et al. utilised a multivariate regression analysis to confirm that a larger tunical defect size exceeding an 18.5 mm cut-off and a delayed time to surgery exceeding 14.5 hours were the most powerful independent predictors of both organic erectile dysfunction and persistent penile curvature [26].

Psychosexual and Psychological Outcomes

Although expert surgical intervention can reliably preserve erectile tissue physiology, the psychological effect of the injury constitutes a prevalent and distinct domain of patient morbidity.

Paradox of Preserved Potency and Psychogenic Distress

The selected studies revealed a notable paradox in which objectively preserved erectile function did not necessarily correspond with satisfactory psychosexual recovery. Many patients demonstrated restored organic erectile capacity on postoperative assessment but continued to experience substantial psychological distress that negatively affected sexual function. Barros et al. prospectively evaluated 58 patients 18 months after surgical repair and found that organic erectile function was preserved in 86.2% of cases [24]. Despite this favourable physiological outcome, 77.5% of patients reported a persistent fear of sustaining another penile fracture during subsequent sexual activity, while 68.9% permanently modified their sexual practices by avoiding vigorous intercourse or abandoning particular sexual positions because of anxiety. Patients who reported performance anxiety were significantly more likely to develop secondary psychogenic erectile dysfunction (p = 0.0337), as were those who perceived a negative impact on their sexual life (p = 0.0418) [24].

Similar findings were reported by Shah et al., who observed that approximately 65% of patients experienced varying degrees of subjective sexual dysfunction despite successful surgical reconstruction and high objective Erection Hardness Scores [26, 30]. In many cases, these symptoms required psychosexual counselling and pharmacological reassurance [26]. Bolat et al. further demonstrated the potentially progressive nature of this burden in a cohort of 64 patients followed for 24 months using the Self-Esteem and Relationship (SEAR) questionnaire [18, 22]. Although erectile-function domain scores remained stable throughout follow-up, mean relationship and self-esteem scores declined significantly over time (p < 0.05). Collectively, these findings suggest that successful anatomical repair and preservation of potency may coexist with persistent fear, altered sexual behaviour, impaired self-esteem, and deterioration in intimate relationships [22].

Contradictory Evidence: The Absence of Broad Psychiatric Morbidity

Although much of the reviewed literature reports postoperative psychosexual distress, some evidence suggests a more favourable psychological recovery profile. Penbegul et al. compared 32 patients who underwent immediate surgical repair of penile fracture with 30 demographically matched healthy controls at a mean follow-up of 15.9 months [20]. Using the Hospital Anxiety and Depression Scale (HADS), Premature Ejaculation Diagnostic Tool (PEDT), and Golombok-Rust Inventory of Sexual Satisfaction (GRISS), the authors found no statistically significant differences between the groups. Mean HADS anxiety scores were 6.4 ± 5.8 in patients and 5.6 ± 2.3 in controls (p = 0.71), while HADS depression scores were 4.6 ± 3.4 and 5.8 ± 3.5, respectively (p = 0.49). Similarly, GRISS and PEDT scores did not differ significantly between groups [20]. These findings suggest that immediate surgical repair is not necessarily associated with broad psychiatric morbidity or adverse psychogenic outcomes in all patients. However, more specific psychosexual effects may not be fully captured by general psychiatric screening tools and may require targeted assessment.

Impact on Ejaculatory Function

The physiological and psychological trauma of a penile fracture also exerts a measurable impact on ejaculatory latency. Penbegul et al. retrospectively analysed ejaculatory parameters and discovered a paradoxical, statistically significant increase in the mean Intravaginal Ejaculation Latency Time (IELT) following penile fracture surgery (p=0.007) [20].

Furthermore, they identified a moderate-to-strong positive correlation between the patient's postoperative Beck Depression Inventory score and the prolonged post-fracture IELT (r = 0.498, p = 0.008). This finding indicates that delayed ejaculation in this population is driven by a dual mechanism: minor, subclinical neurogenic damage to the dorsal nerve branches sustained during the traumatic hyperflexion, which is severely compounded by the cognitive distraction, depression, and localised anxiety experienced by the patient during intercourse.

Discussion

The systematic synthesis of the nine core studies in this scoping review confirms that immediate surgical repair remains the absolute, unquestioned gold standard of care for preserving organic erectile function and anatomical alignment following a penile fracture. The meta-analytical data compiled by Abdelrasheed et al. and the multicentre European data from Bozzini et al. confirm that surgical intervention within 24 hours and ideally within the first eight hours drastically reduces the incidence of organic erectile dysfunction and severe penile curvature [4, 23]. Early intervention achieves this by evacuating the organising haematoma, thereby preventing smooth muscle ischaemia and the subsequent deposition of inelastic collagen within the cavernosal tissue [4, 23].

However, analysis of psychosexual parameters shows a significant gap in the standard urological definition of "clinical success." In clinical practice, recovery is often defined by a straight erection and an IIEF-5 score above 21. Nevertheless, long-term data from Barros et al. and the 24-month cohort of Bolat et al. indicate that psychological sequelae persist beyond anatomical healing [22, 24]. The high prevalence of fear of recurrence (77.5%) and permanent changes in sexual practices (68.9%) suggests that many patients experience chronic sympathetic nervous system arousal during intimacy [22]. This sympathetic activation opposes the parasympathetic nitric oxide pathway required for corporal relaxation, resulting in severe psychogenic erectile dysfunction even when Doppler ultrasound demonstrates normal cavernosal hemodynamics.

Furthermore, the controlled psychometric data from Penbegul et al. suggest that urologists cannot rely on generalised depression or anxiety screening tools, such as the HADS or Beck Depression Inventory, to identify these struggling patients [20]. Because the trauma is highly context-specific, broad scales will often fail to detect the localised performance anxiety and relationship degradation that Bolat et al. and Shah et al. identified as progressively worsening over time [22, 26].

To optimise long-term recovery, postoperative follow-up should include assessment of erectile function, sexual confidence, and psychosexual well-being. Pharmacological support, including phosphodiesterase type 5 (PDE-5) inhibitors, may be considered for patients with clinically evident postoperative erectile dysfunction, but routine use after penile fracture repair requires further evaluation. Educating patients about the physical strength of the repaired tunica albuginea and employing sensate focus techniques may help dissociate intimacy from fear, enabling parasympathetic regulation of erection without interference from trauma-induced sympathetic activation.

Limitations

Several limitations within the synthesised primary literature should be acknowledged. First, aside from the prospective cohorts by Barros et al. and the controlled design of Penbegul et al., most selected studies are retrospective reviews, which are inherently susceptible to selection, recall, and information biases [20, 24]. Patients are often required to retrospectively recall their pre-injury sexual baseline, possibly causing an overestimation of pre-fracture potency.

Second, there is a lack of standardised, trauma-specific psychometric instruments in urological follow-up protocols. The studies evaluated in this review utilised a heterogeneous array of scales (IIEF-5, SEAR, HADS, GRISS, PEDT, and the Beck Depression Inventory), which prevents the execution of a robust, pooled quantitative meta-analysis of the psychosexual data.

Finally, the psychological experience of female partners is entirely unrepresented in the current literature, representing a significant gap given that relationship satisfaction scores and sexual frequency have been shown to decline progressively over a 24-month follow-up period.

This review was guided by a predefined protocol; however, the protocol was not prospectively registered in a public database. This may limit methodological transparency and should be considered when interpreting the findings.

## Conclusions

The contemporary surgical management of penile fracture is highly effective in preserving anatomical form and organic erectile function in most patients. Evidence from this scoping review supports immediate surgical repair, ideally within 24 hours of injury, particularly in patients with high-risk anatomical features such as advanced age, large tunical tears, bilateral corporal involvement, and concomitant urethral injury.

However, clinical success should not be judged solely by structural or haemodynamic outcomes. The psychosexual consequences of penile fracture may persist even after successful anatomical repair, with some patients experiencing performance anxiety, fear of reinjury, avoidance behaviours, reduced self-esteem, and relationship-related concerns. To support broader recovery, postoperative follow-up should include assessment of erectile function, psychosexual well-being, and relationship-related outcomes. Where clinically indicated, patients with postoperative erectile dysfunction may benefit from appropriate sexual health support. Still, specific interventions such as routine PDE-5 inhibitor rehabilitation require further evaluation before they can be recommended as standard postoperative care.

## Appendices

**Table 4:** Outcome domains and assessment tools ED: erectile dysfunction; PDE-5: phosphodiesterase type 5; IIEF-5: International Index of Erectile Function-5; SHIM: Sexual Health Inventory for Men; PEDT: Premature Ejaculation Diagnostic Tool; HADS: Hospital Anxiety and Depression Scale; BDI: Beck Depression Inventory; GRISS: Golombok-Rust Inventory of Sexual Satisfaction; SEAR: Self-Esteem and Relationship Questionnaire

Outcome domain	Components assessed	Common tools/measures
Anatomical outcomes	Penile curvature, deformity, tunical scarring, palpable plaques or nodules, pain, urethral injury, wound complications	Clinical examination, operative findings, follow-up assessment, patient-reported pain or deformity
Erectile function/ED	Ability to achieve and maintain erection, ED severity, erectile rigidity, need for PDE-5 inhibitors	IIEF-5, SHIM, Erection Hardness Score, reported ED rates
Broader sexual function	Intercourse satisfaction, overall sexual satisfaction, resumption of sexual activity, painful erections, ejaculation, ejaculatory latency	IIEF domains, PEDT, intravaginal ejaculatory latency time, patient-reported sexual satisfaction
Psychological outcomes	Fear of recurrence, performance anxiety, psychogenic ED, anxiety symptoms, depressive symptoms, sexual confidence	HADS, Beck Depression Inventory, study-specific psychosexual questionnaires
Relationship outcomes	Relationship satisfaction, partner-related concerns, self-esteem, avoidance of intercourse, changes in sexual habits or sexual positions	GRISS, SEAR questionnaire, patient-reported relationship or partner satisfaction outcomes
